# The Transient Receptor Potential Vanilloid 4 Channel and Cardiovascular Disease Risk Factors

**DOI:** 10.3389/fphys.2021.728979

**Published:** 2021-09-20

**Authors:** Kenichi Goto, Takanari Kitazono

**Affiliations:** ^1^Department of Health Sciences, Graduate School of Medical Sciences, Kyushu University, Fukuoka, Japan; ^2^Department of Medicine and Clinical Science, Graduate School of Medical Sciences, Kyushu University, Fukuoka, Japan

**Keywords:** endothelial dysfunction, transient receptor potential vanilloid type 4 channel, endothelium-dependent hyperpolarization, nitric oxide, hypertension, obesity, diabetes mellitus, aging

## Abstract

Vascular endothelial cells regulate arterial tone through the release of nitric oxide and other diffusible factors such as prostacyclin and endothelium derived hyperpolarizing factors. Alongside these diffusible factors, contact-mediated electrical propagation from endothelial cells to smooth muscle cells *via* myoendothelial gap junctions, termed endothelium-dependent hyperpolarization (EDH), plays a critical role in endothelium-dependent vasodilation in certain vascular beds. A rise in intracellular Ca^2+^ concentration in endothelial cells is a prerequisite for both the production of diffusible factors and the generation of EDH, and Ca^2+^ influx through the endothelial transient receptor potential vanilloid 4 (TRPV4) ion channel, a nonselective cation channel of the TRP family, plays a critical role in this process in various vascular beds. Emerging evidence suggests that the dysregulation of endothelial TRPV4 channels underpins endothelial dysfunction associated with cardiovascular disease (CVD) risk factors, including hypertension, obesity, diabetes, and aging. Because endothelial dysfunction is a precursor to CVD, a better understanding of the mechanisms underlying impaired TRPV4 channels could lead to novel therapeutic strategies for CVD prevention. In this mini review, we present the current knowledge of the pathophysiological changes in endothelial TRPV4 channels associated with CVD risk factors, and then explore the underlying mechanisms involved.

## Introduction

**Figure 1:**
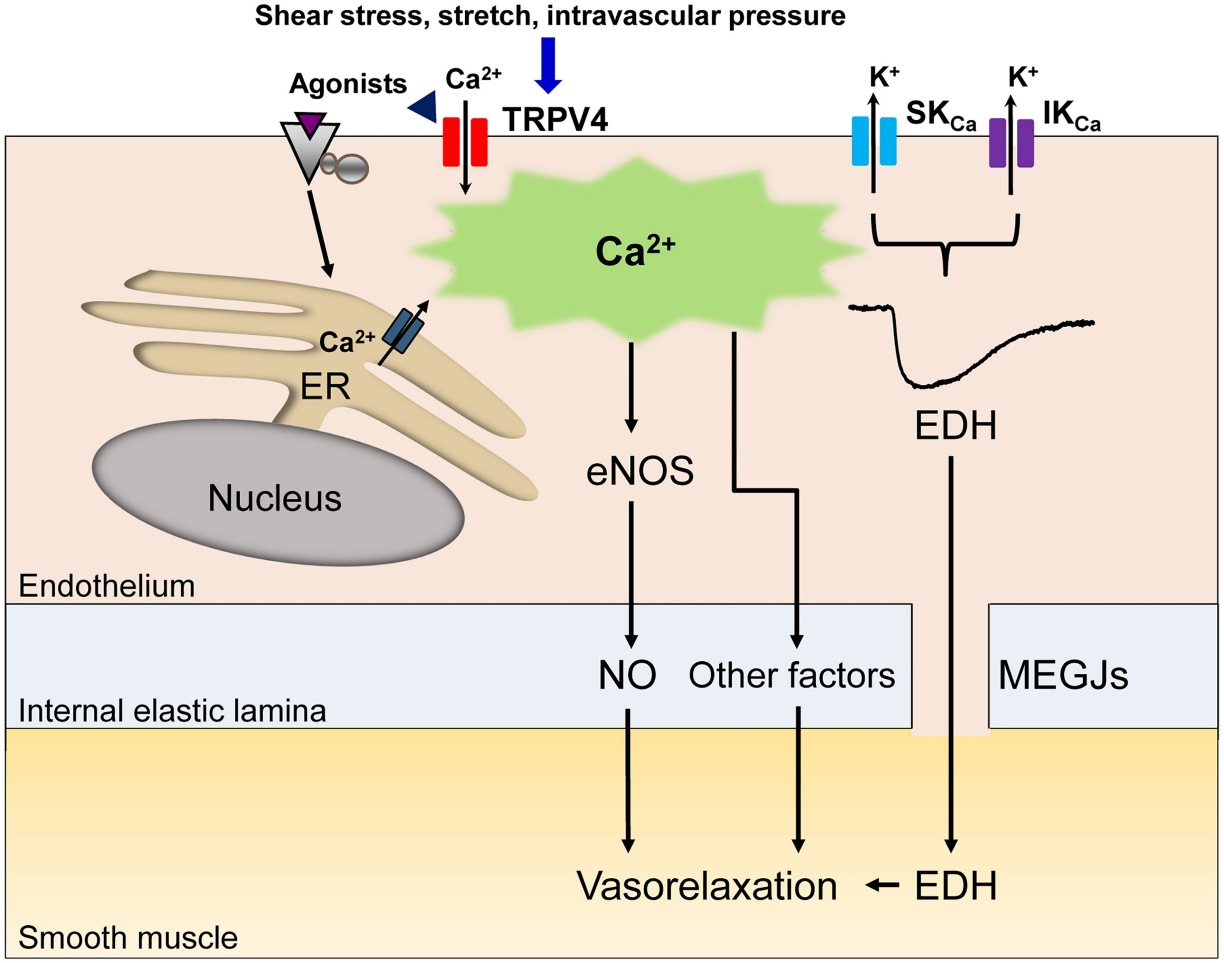
A schematic diagram of endothelial Ca^2+^ signaling-dependent vasorelaxation. Endothelial stimulation with agonists, shear stress, stretch or intravascular pressure increases the intracellular Ca^2+^ concentration due to Ca^2+^ release from the endoplasmic reticulum (ER) and/or Ca^2+^ influx through endothelial nonselective cation channels of the transient receptor potential vanilloid type 4 (TRPV4). The rise in the endothelial Ca^2+^ concentration elicits the release of endothelial nitric oxide (NO) and other vasodilatory mediators. In specific vascular beds, the rise in the endothelial Ca^2+^ concentration subsequently activates small (SK_Ca_) and intermediate conductance (IK_Ca_) Ca^2+^-activated K^+^ channels, generating endothelium-dependent hyperpolarization (EDH), which spreads to adjacent smooth muscle cells *via* myoendothelial gap junctions (MEGJs), leading to vasorelaxation.

In vascular endothelial cells, a rise in intracellular Ca^2+^ following agonist and shear stress stimulation causes vasorelaxation through the generation of nitric oxide (NO; Vanhoutte et al., 2017; Goto et al., 2018). In some vascular beds, other diffusible factors including epoxyeicosatrienoic acid (EET), potassium ions, hydrogen peroxide and prostacyclin contribute to endothelium-dependent vasorelaxation (Vanhoutte et al., 2017; Goto et al., 2018). Alongside these diffusible factors, a rise in intracellular Ca^2+^ in endothelial cells generates endothelium-dependent hyperpolarization (EDH) through the activation of endothelial small (SK_Ca_) and intermediate conductance (IK_Ca_) Ca^2+^-activated K^+^ channels, which spreads to adjacent smooth muscle cells *via* myoendothelial gap junctions (MEGJs) and causes vasorelaxation in many vascular beds (Goto et al., 2018; Murphy and Sandow, 2019; Figure 1). Thus, dysregulation of this Ca^2+^ rise in vascular endothelial cells could lead to a loss of endothelium-dependent vasorelaxation, thereby inducing endothelial dysfunction.

A transient rise in endothelial Ca^2+^ following physiological stimuli depends on the release of Ca^2+^ from the endoplasmic reticulum and on Ca^2+^ influx across the plasma membrane of endothelial cells. In addition, accumulating evidence suggests that Ca^2+^ influx in the endothelial cells of various vascular beds is mediated by nonselective cation channels of the transient receptor potential (TRP) family (Sullivan and Earley, 2013; Hill-Eubanks et al., 2014; Earley and Brayden, 2015) and the Ca^2+^ influx through some of these TRP channels, i.e., TRP vanilloid 1 (TRPV1), TRPV3, TRPV4, TRP ankyrin 1 (TRPA1), TRP canonical 3 (TRPC3), and TRPC4 has been reported to play an important role in endothelium-dependent vasorelaxation in certain vascular beds (Earley and Brayden, 2015).

Among these TRP channels, the TRPV4 channel has been receiving extensive attention in cardiovascular physiology because of its wide expression in vascular endothelial cells and its relatively high Ca^2+^ permeability (Filosa et al., 2013; Hill-Eubanks et al., 2014; Heathcote et al., 2019; Chen and Sonkusare, 2020). Such properties make TRPV4 highly important for endothelium-dependent vasorelaxation in response to physiological stimuli such as shear stress (Köhler et al., 2006), EET (Earley et al., 2009), acetylcholine (Zhang et al., 2009; Sonkusare et al., 2012), adenosine triphosphate (Marziano et al., 2017), stretch (Thodeti et al., 2009), and intravascular pressure (Bagher et al., 2012) in specific vascular beds under specific conditions. Moreover, increasing evidence reveals that endothelial TRPV4 are involved in the pathophysiological process of endothelial dysfunction in certain vascular beds in cardiometabolic disease conditions (Chen and Sonkusare, 2020).

This mini review therefore focuses on the pathophysiological role of the TRPV4 channel in endothelial dysfunction associated with cardiovascular disease (CVD) risk factors such as hypertension, obesity, diabetes mellitus and ageing, along with its underlying mechanisms (Table 1).

**Table 1:** Changes in TRPV4 expression and function in animal models of diseases.

Diseases	Species/Sex	Animals	Vascular bed	Agonist	Outcome on TRPV4 expression and function	References
Genetic hypertension	Rat/male	20weeks old SHRSP	Mesenteric	GSK	Reduced TRPV4 expression and function	Seki et al., 2017
	Rat/male	10–12weeks old SHR	Mesenteric	4αPDD	Reduced TRPV4 expression and function	Boudaka et al., 2019
Ang II-induced hypertension	C57BL/6 mice/male	4weeks treatment with Ang II	Parenchymal arterioles	GSK	Reduced TRPV4 expression and function	Diaz-Otero et al., 2018
	C57BL/6 mice/male	2weeks treatment with Ang II	Mesenteric	GSK	Reduced TRPV4 function with no altered expression	Nishijima et al., 2014
	C57BL/6 mice/male	3weeks treatment with Ang II	Mesenteric	GSK	Reduced TRPV4 function with no altered expression	Sonkusare et al., 2014
Salt-induced hypertension	C57BL/6 mice	8% NaCl for 3weeks	Mesenteric	GSK	Impaired functional interaction of TRPV4 and SK_Ca_ with no altered expression	He et al., 2017
	Rat/male	4% NaCl for 3weeks	Mesenteric	4αPDD	Increased TRPV4 expression and function	Gao et al., 2009
	Mice/male	8% NaCl for >3weeks	Aorta	GSK	Increased TRPV4 induced vasoconstriction with no altered expression	Zhang et al., 2018
Obesity	C57BL/6 mice/male	60% of total energy from fat for 14weeks	Mesenteric (third-order)	GSK	Reduced TRPV4 function due to the result of increased peroxynitrite formation	Ottolini et al., 2020
	C57BL/6 mice/male	60% of total energy from fat for 21weeks	Mesenteric (third-order)	GSK	Preserved TRPV4 function	Greenstein et al., 2020
Diabetes	Rat/male	12–15weeks after STZ treatment	Mesenteric	4αPDD	Reduced TRPV4 expression and function	Ma et al., 2013
	Rat/male	3months after STZ treatment	Retinal arteriole	ND	Reduced TRPV4 expression	Monaghan et al., 2015
	Rat/male	8–14days after STZ treatment	Aorta	4αPDD	Reduced TRPV4 expression and function	Shamsaldeen et al., 2020
	C57BL/6 mice/male	4months after STZ treatment	Aorta	Vildagliptin	Reduced TRPV4 expression and function	Gao et al., 2020
Aging	Rat	22months old rats	Mesenteric	4αPDD	Reduced TRPV4 expression and function	Du et al., 2016
	Rat/male	19–21months old Sprague-Dawley rats	Aorta	GSK	Reduced TRPV4 function	Huang et al., 2019

Ang II, angiotensin II; GSK, GSK1016790A; SK_Ca_, small-conductance Ca^2+^-activated K^+^ channel; SHR, spontaneously hypertensive rats; SHRSP, stroke-prone spontaneously hypertensive rats; STZ, streptozotocin; TRPV4, transient receptor potential vanilloid 4 channel.

## TRPV4 and Hypertension

### Genetic Hypertension

While a reduction in NO bioavailability and/or enhanced production of endothelium-derived contracting factors are generally recognized as contributing to endothelial dysfunction during hypertension (Goto et al., 2018), we have shown that the impairment of EDH-mediated responses contributes greatly to endothelial dysfunction in genetically hypertensive rats (Goto et al., 2004, 2018).

In our 2017 paper, we showed that the opening of endothelial TRPV4 and the subsequent activation of both SK_Ca_ and IK_Ca_ channels are prerequisites for the generation of EDH in the superior mesenteric arteries of normotensive Wistar-Kyoto (WKY) rats (Seki et al., 2017). Even more importantly, we demonstrated that reduced expression and function of both endothelial TRPV4 and SK_Ca_ underpinned the impaired EDH-mediated responses in superior mesenteric arteries of 20-week-old stroke-prone spontaneously hypertensive rats (SHRSP; Seki et al., 2017).

In accord with our findings, Boudaka et al. (2019) reported decreased expression of endothelial TRPV4 and impaired endothelium-dependent vasorelaxation in second-order branches of the mesenteric arteries of 10- to 12-week-old SHR, although some disparities exist between the two studies. While the downregulation of endothelial TRPV4 led to a loss of NO in the Boudaka et al. (2019) study, the downregulation of endothelial TRPV4 led to a loss of EDH in our study. The reason for the disparity is not clear, but it might be related to the differences in strain, age, or vessel diameter used. For instance, in rat superior mesenteric arteries, the relative contribution of EDH in endothelium-dependent vasorelaxation is more apparent in the distal segment of this artery (Simonsen et al., 1999; Stankevicius et al., 2011). Nevertheless, what should be noted here is that both studies highlight endothelial TRPV4 as a key factor contributing to the endothelial dysfunction in genetic hypertension.

Caveolae, which are specialized lipid rafts that form flask-shaped invaginations of the plasma membrane, are particularly abundant in vascular endothelial cells, and many signal transduction proteins localized in caveolae play important roles in endothelial cell signaling and function (Sowa, 2012). While it is well-documented that endothelial nitric oxide synthase (eNOS) localizes in caveolae (Sowa, 2012), several recent studies have also demonstrated that both TRPV4 and SK_Ca_ preferentially co-localize and functionally interact in the caveolae of vascular endothelial cells (Goedicke-Fritz et al., 2015; Lu et al., 2017). Moreover, the disruption of caveolae impaired NO and/or EDH-mediated vasorelaxations in certain vascular beds (Xu et al., 2007).

In contrast to SK_Ca_, endothelial IK_Ca_ are thought to reside in lipid rafts outside caveolae (Absi et al., 2007; Goedicke-Fritz et al., 2015). In this context, it is of particular interest that the number of endothelial caveolae quantified by electron microscopy was significantly decreased in the aortas and mesenteric arteries of SHRs compared to those of WKY rats (Potje et al., 2019). The decrease in the number of endothelial caveolae appears not to have been strain-specific, but rather to be due to elevated blood pressure, because a decreased number of caveolae in endothelial cells has also been reported in renal hypertensive (2K-1C) rat aortas (Rodrigues et al., 2010). Further support for this notion comes from the observation that increases in intravascular pressure, associated with the disassembly of caveola structural proteins caveolin-1 and cavin-1, corresponded with a decreased number of caveolae on the plasma membrane of carotid endothelial cells (Michell et al., 2021). Taking these results together, it is intriguing to speculate whether decreases in the number of endothelial caveolae during hypertension lead to a loss of both TRPV4 and SK_Ca_ in endothelial cells, which in turn impairs the endothelium-dependent, NO- and/or EDH-mediated vasorelaxation in genetic hypertension.

### Angiotensin II-Induced Hypertension

In parenchymal arterioles of mice with angiotensin II (AngII)-induced hypertension, the reduced expression and function of both TRPV4 and SK_Ca_ underpin impaired carbachol- and TRPV4 agonist-induced (probably EDH-mediated) vasorelaxation (Diaz-Otero et al., 2018). These results were similar to those found in mesenteric arteries of SHRSP (Seki et al., 2017). Of interest here is the observation that these mice had elevated plasma aldosterone levels, and administration of the selective mineralocorticoid receptor antagonist eplerenone restored the reduced expression and function of both TRPV4 and SK_Ca_ without affecting blood pressure levels (Diaz-Otero et al., 2018; Chambers and Dorance, 2020). These findings suggest that aldosterone downregulates the expression of TRPV4 and SK_Ca_ in parenchymal arterioles during Ang II-induced hypertension. Because plasma aldosterone levels are also elevated in SHRSP (Kim et al., 1992), it is worth investigating whether aldosterone contributes to impaired EDH in SHRSP through its modulation of TRPV4 and/or SK_Ca_.

An earlier study of mice with AngII-induced hypertension likewise showed impairment of ACh- and TRPV4 agonist-induced vasorelaxation mechanisms in the mesenteric arteries (Nishijima et al., 2014). However, in contrast to the results seen in the parenchymal arterioles, the expression of TRPV4 themselves was not reduced in the mesenteric arteries of AngII-induced hypertensive mice (Nishijima et al., 2014). The reason for the difference in the expression of TRPV4 between the two studies is unclear: there was a difference in the duration of the Ang II treatment period (4weeks in the study by Diaz-Otero et al., 2018 vs. 2weeks in the study by Nishijima et al., 2014), or the effect of Ang II on TRPV4 expression might differ from one vessel to the other.

Indeed, in yet another study of AngII-induced hypertensive mice, carbachol- and TRPV4 agonist-induced, EDH-mediated vasorelaxations in mesenteric arteries were impaired due to reduced TRPV4 channel activity with no change in the number of TRPV4 (Sonkusare et al., 2014). In some vascular beds including mice mesenteric arteries, TRPV4 and IK_Ca_ are co-localized to myoendothelial projections (MEPs) and cooperative activation of TRPV4 and IK_Ca_ at these sites evokes EDH (Bagher et al., 2012; Sonkusare et al., 2014; Ellinsworth et al., 2016; Murphy and Sandow, 2019). In mesenteric arteries of AngII-induced hypertensive mice, Ang II reduced the levels of regulatory protein A-kinase anchoring protein 150 (AKAP150) at MEPs, leading to the impairment of both muscarinic receptor-PKC activation of TRPV4 and AKAP150-dependent clustering of TRPV4 (Sonkusare et al., 2014; Chen and Sonkusare, 2020). The resulting decrease in Ca^2+^ influx through compromised TRPV4 at MEPs in this model disrupts the downstream activation of IK_Ca_, leading to the loss of EDH.

To sum up, although these studies suggest a causal link between the TRPV4 and endothelial dysfunction during Angiotensin II-induced hypertension, the underlying mechanisms seem to be multiple and warrant further investigation.

### Salt-Induced Hypertension

In the mesenteric arteries of hypertensive mice fed a high-salt diet (8% NaCl for 3weeks), ACh- and TRPV4 agonist-induced vasorelaxations were impaired principally due to reduced EDH activity. This impairment was the result of compromised physical and functional interaction of TRPV4 and SK_Ca_ at endothelial caveolae but was not associated with reduced expressions of these channels (He et al., 2017).

The effect of a high-salt diet on vascular TRPV4 channel regulation, however, appears to vary depending on species and the vascular bed studied; in the mesenteric arteries of rats, a high-salt diet (4% NaCl for 3weeks) increased TRPV4 expression and augmented TRPV4-induced blood pressure lowering (Gao et al., 2009), suggesting that TRPV4 may be upregulated to maintain endothelial function and blood pressure during salt-induced hypertension. This hypothesis agrees well with our previous findings that a high-salt diet (8% NaCl for 6weeks) upregulated EDH to compensate for the loss of NO in the mesenteric arteries of rats (Goto et al., 2012).

Intriguingly, a recent report by Zhang et al. (2018) showed that in aortas of hypertensive mice fed a high-salt diet (8% NaCl for >3weeks), Ca^2+^ influx through TRPV4 induced endothelium-dependent vasoconstriction probably due to the increased activation of the cytosolic phospholipase A2/cyclooxygenase-2 (COX2)/prostaglandin F_2α_ signaling pathway, followed by increased expression of COX2.

Nevertheless, since the aorta is a conduit vessel that does not play an important role in the regulation of vascular resistance, further investigations are needed to examine whether TRPV4 channel-mediated, endothelium-dependent vasoconstriction is also present in resistance arteries, and if so, whether such a mechanism contributes to the impairment of endothelial function in salt-induced hypertension.

## TRPV4 and Obesity

The accumulation of both visceral and perivascular fat upregulates secretion of inflammatory cytokines and generation of ROS in the vasculature, thereby leading to endothelial dysfunction in obesity (Goto and Kitazono, 2020).

In this respect, a recent study by Ottolini et al. (2020) is highly interesting. In that study, they showed that both carbacol- and TRPV4 agonist-induced vasorelaxations were impaired in the third-order mesenteric arteries of male C57BL6/J mice with diet-induced (60% of total energy from fat for 14weeks) obesity (Ottolini et al., 2020); this was the result of increased peroxynitrite formation at MEPs, which in turn oxidized the regulatory protein AKAP150 to impair AKAP150-dependent cooperative TRPV4 activation without altering TRPV4 expression (Ottolini et al., 2020). Of note, the increased peroxynitrite formation also impaired endothelial TRPV4 activities and thus TRPV4-mediated vasorelaxation in the splenius and temporalis muscle arteries of obese individuals (Ottolini et al., 2020).

However, in sharp contrast to the results of Ottolini et al. (2020), Greenstein et al. (2020) reported that in the third-order mesenteric arteries of male C57BL6/J mice fed a high-fat diet (60% of total energy from fat for 21weeks), an almost identical experimental protocol as used by Ottolini’s group, neither carbacol- nor TRPV4 agonist-induced vasorelaxation was impaired, suggesting that the activity of the endothelial TRPV4 was preserved. Instead, Greenstein et al. (2020) showed enhanced vascular tone due to impaired large-conductance Ca^2+^-activated potassium channel function in vascular smooth muscle cells.

The reason for the discrepancies between the two studies despite the nearly identical experimental protocols is not known. Differences in duration of diet, changes in the microbiome or genetic drift of the mice might explain such discrepancies as Fulton and Stepp (2020) have pointed out. Alternatively, differences in mean blood pressure in the two studies (around 110–125mmHg in the study by Ottolini et al., 2019, 2020 vs. around 90mmHg in the study by Greenstein et al., 2020) might account for the different results. Indeed, it has been reported that blood pressure levels positively correlate with some oxidative stress-related parameters in hypertension (Rodrigo et al., 2007), and the higher levels of blood pressure achieved in the study by Ottolini et al. (2019, 2020) might preferentially lead to an increased peroxynitrite formation at MEPs and hence impaired activity of TRPV4.

It is thus apparent that the pathophysiological relevance of TRPV4 to endothelial dysfunction associated with obesity is yet to be determined and warrants further investigation.

## TRPV4 and Diabetes

Endothelial dysfunction, a common feature of diabetes, is closely associated with diabetic vascular complications (Goto and Kitazono, 2019). With respect to the link between the TRPV4 and diabetes-induced endothelial dysfunction, a high-glucose medium downregulated the protein expression of TRPV4 and attenuated the agonist-stimulated Ca^2+^ influx in both bovine retinal microvascular endothelial cells and human umbilical vein endothelial cells (Monaghan et al., 2015; Gao et al., 2020).

Moreover, in blood vessels from animal models of diabetes, reduced TRPV4 expression has been consistently reported across numerous studies. These blood vessels include mesenteric arteries (Ma et al., 2013), retinal arterioles (Monaghan et al., 2015), and aortas (Shamsaldeen et al., 2020) of streptozotocin (STZ)-induced diabetic rats, as well as in aortas of STZ-induced diabetic mice and db/db mice (Gao et al., 2020). Furthermore, the reduction in TRPV4 expression was consistently associated with a decrease in agonist-stimulated Ca^2+^ influx in endothelial cells, which led to an impairment of either NO (Gao et al., 2020; Shamsaldeen et al., 2020)- or EDH (Ma et al., 2013)-mediated vasorelaxation, depending on the vascular bed studied. Taken together, these findings strongly indicate that downregulation of TRPV4 expression contributes to the endothelial dysfunction associated with diabetes.

Although the underlying mechanism of TRPV4 downregulation in diabetes is not known, a recent finding by Shamsaldeen et al. (2020) may provide a clue. In the aortas of STZ-induced diabetic rats with endothelial dysfunction, the reduction in TRPV4 expression was accompanied by a reduction in both eNOS and caveolin-1 expression; insulin treatment reversed the endothelial dysfunction and was associated with the upregulation of TRPV4 expression, eNOS and caveolin-1 (Shamsaldeen et al., 2020).

Given this finding, together with the observation that TRPV4, eNOS and caveolin-1 are preferentially co-localized and functionally interactive in the caveolae of certain vascular endothelial cells (Sowa, 2012; Goedicke-Fritz et al., 2015), it is possible to hypothesize that a decrease in the number of endothelial caveolae is causally connected to the downregulation of endothelial TRPV4 expression and thus to the endothelial dysfunction in diabetes. In fact, the number of endothelial caveolae quantified by electron microscopy was significantly decreased in diabetic patients with endothelial dysfunction, possibly due to the disruption of caveolae by peroxynitrite (Cassuto et al., 2014). Glucose lowering by insulin might prevent the caveolae disruption.

## TRPV4 and Aging

In the mesenteric arteries of aged (22-month-old) rats, TRPV4 agonist- and flow-induced vasorelaxations were reduced compared with those in young (3-month-old) rats (Du et al., 2016). These age-related reductions were associated with reduced expression of endothelial TRPV4 and lowered Ca^2+^ influx through TRPV4 (Du et al., 2016). Because increasing the TRPV4 expression using gene delivery by lentiviral vectors restored the reduced TRPV4 agonist- and flow-induced vasorelaxations in aged rats, reduced expression of endothelial TRPV4 appears causally related to the age-associated endothelial dysfunction in this model (Du et al., 2016).

Reduced TRPV4 agonist-induced vasorelaxation, possibly due to impaired TRPV4-SK_Ca_ signaling, was reported in the thoracic aortas of aged (19- to 21-month-old) Sprague Dawley rats compared with that in young (2-month-old) rats (Huang et al., 2019). Interestingly, 3months of consistent exercise reversed the age-related impairment of the TRPV4-mediated vasorelaxation, although the mechanisms underlying the restoration of function remain unknown (Huang et al., 2019).

## TRPV4 and Other Factors

While a few studies suggest that sex (Wong et al., 2015) and hyperlipidemia (Matsumoto et al., 2017) affect TRPV4 signaling in specific vascular beds, many aspects remain unclear and warrant further investigations.

In addition, functional TRPV4 is also present in smooth muscle cells of some vascular beds (Filosa et al., 2013; Earley and Brayden, 2015; Ottolini et al., 2019) and several physiological stimuli including EET (Earley et al., 2009) and stretch (Gebremedhin et al., 2017) cooperatively activate smooth muscle TRPV4 and K_Ca_ channels in certain vascular beds. Thus, dysregulation of smooth muscle TRPV4 might also be related to the various results described in this mini review.

## Conclusion and Clinical Perspective

The studies reviewed in this paper indicate that dysregulation of endothelial TRPV4 contributes to the endothelial dysfunction associated with CVD risk factors, such as hypertension, obesity, diabetes, and aging in animal models of diseases.

In humans, functional endothelial TRPV4 are expressed in cerebral (Hatano et al., 2013), coronary (Bubolz et al., 2012; Zheng et al., 2013; Cao et al., 2018), and microvasculature (Caires et al., 2017; Goedicke-Fritz et al., 2015). It is thus intriguing to speculate that impairment of TRPV4 may as well underpin endothelial dysfunction in hypertensive, obese, diabetic and elderly individuals. Since endothelial dysfunction is a precursor to CVD, establishing therapeutic strategies targeting endothelial TRPV4 could be of clinical importance.

Direct activation of endothelial TRPV4 may be a possible strategy. However, caution should be exercised in this approach as TRPV4 activation leads to hypotension and vascular failure (Simonsen et al., 2017). Blockade of renin angiotensin aldosterone system and scavenging of peroxynitrite may be beneficial for specific disease conditions.

Better understanding of the pathophysiology of the dysregulation of endothelial TRPV4 associated with CVD risk factors will very likely open new avenues for the prevention and treatment of CVD.

## Author Contributions

All authors listed have made a substantial, direct and intellectual contribution to the work, and approved it for publication.

## Conflict of Interest

The authors declare that the research was conducted in the absence of any commercial or financial relationships that could be construed as a potential conflict of interest.

## Publisher’s Note

All claims expressed in this article are solely those of the authors and do not necessarily represent those of their affiliated organizations, or those of the publisher, the editors and the reviewers. Any product that may be evaluated in this article, or claim that may be made by its manufacturer, is not guaranteed or endorsed by the publisher.
